# Material Extrusion Additive Manufacturing of Wood and Lignocellulosic Filled Composites

**DOI:** 10.3390/polym12092115

**Published:** 2020-09-17

**Authors:** Meghan E. Lamm, Lu Wang, Vidya Kishore, Halil Tekinalp, Vlastimil Kunc, Jinwu Wang, Douglas J. Gardner, Soydan Ozcan

**Affiliations:** 1 Manufacturing Demonstration Facility, Energy and Transportation Science Division, Oak Ridge National Laboratory, 2350 Cherahala Boulevard, Knoxville, TN 37932, USA; kishorev@ornl.gov (V.K.); tekinalphl@ornl.gov (H.T.); kuncv@ornl.gov (V.K.); 2 Advanced Structures and Composites Center, University of Maine, 35 Flagstaff Road, Orono, ME 04469, USA; lu.wang@maine.edu (L.W.); douglasg@maine.edu (D.J.G.); 3 School of Forest Resources, University of Maine, 5755 Nutting Hall, Orono, ME 04469, USA; 4 Forest Products Laboratory, U.S. Forest Service, 1 Gifford Pinchot Drive, Madison, WI 53726, USA; jinwu.wang@usda.gov

**Keywords:** materials extrusion, lignocellulosic biomass, wood composites, additive manufacturing, 3D-printing)

## Abstract

Wood and lignocellulosic-based material components are explored in this review as functional additives and reinforcements in composites for extrusion-based additive manufacturing (AM) or 3D printing. The motivation for using these sustainable alternatives in 3D printing includes enhancing material properties of the resulting printed parts, while providing a green alternative to carbon or glass filled polymer matrices, all at reduced material costs. Previous review articles on this topic have focused only on introducing the use of natural fillers with material extrusion AM and discussion of their subsequent material properties. This review not only discusses the present state of materials extrusion AM using natural filler-based composites but will also fill in the knowledge gap regarding state-of-the-art applications of these materials. Emphasis will also be placed on addressing the challenges associated with 3D printing using these materials, including use with large-scale manufacturing, while providing insight to overcome these issues in the future.

## 1. Background

Additive manufacturing (AM) or 3D printing are among the most exciting advances in materials development over the past several decades [1]. The manufacturing space for 3D printing is quite broad with a wide variety of materials that can be utilized including plastics, metals, gels, foams, etc. There are a few common AM manufacturing processes for polymeric materials, including material extrusion processing, liquid deposition modeling, and granular material binding (selective laser sintering). 3D printing is a green manufacturing technique, utilized for a wide range of applications with minimal waste and requiring minimal supervision. These benefits allow for 3D printing with high component customizability and lower production costs for certain applications. The scale of 3D printing processes has historically focused on the bench scale, in which part build volumes are typically around 0.3 to 1 cubic meter. Recently, large scale AM processes have been explored with build volumes of 90 cubic meters and larger that covers the product space of automobile manufacturing and construction applications [2,3].

There is an increasing demand from consumers, industry, and governments on products made from renewable and sustainable resources [4]. For example, many industries are investigating the use of biomass sourced nylons to replace its petroleum-derived analogue [5]. There is also an emphasis on use of materials that are biodegradable, non-petroleum based, carbon neutral, and with low environmental, human health, and safety risks [6,7]. Traditional AM techniques exploit petroleum-based composites which utilize fillers such as carbon fiber and glass materials. Utilization of renewable wood and lignocellulosic-based filler components in polymer composites for AM processes addresses these challenges. In addition, AM of wood and lignocellulosic-based composites have also led to improved material properties for a diverse variety of product applications, making them even more alluring [8,9,10,11].

Previous reviews have provided analysis of materials extrusion AM processing using natural fillers and biopolymers, including lignocellulosic compounds, wood materials, grasses, and other natural fibers [10,12,13,14]. For example, both Mazzanti et al. and Wang et al. provided an in-depth analysis into currently available natural fillers and their subsequent mechanical properties, but both provided only a brief analysis of challenges and outlook [10,13]. Chaunier et al. provided a review focusing on the challenges and outlook associated with processing these materials but did not include much exploration into applications. Liu et al. presented an extensive review on the use of biopolymer fillers in materials extruded AM samples, with a focus on their use in biomedical applications, but did not analyze many other applications for composite materials [14]. While each of these reviews have provided an in-depth discussion into material availability, properties, and even potential challenges, with a focus on some specialized applications, no review has yet to analyze two key state-of-the-art areas of this application: large-scale AM processing and wide-ranging material applications. While lab scale usage and printing with natural fillers is promising as it presents great potential to replace petroleum-based fillers, such as carbon and glass fibers, researchers also need to be able to translate this work towards large-scale designs, which includes both direct and indirect applications that have an impactful effect on industrial manufacturing. Previous issues with materials cost, availability, and properties have limited these thrusts in the past, but new research has provided a multitude of potential routes to success. It is the purpose of this paper to review the current research activities in the use of wood and lignocellulosic-based materials in extrusion AM processes, with an emphasis on these underrepresented areas. Discussion will also include material properties of these composites, manufacturing and processing challenges, and material considerations.

## 2. Additive Manufacturing Overview

### 3D Printing Processes

**Figure 1:**
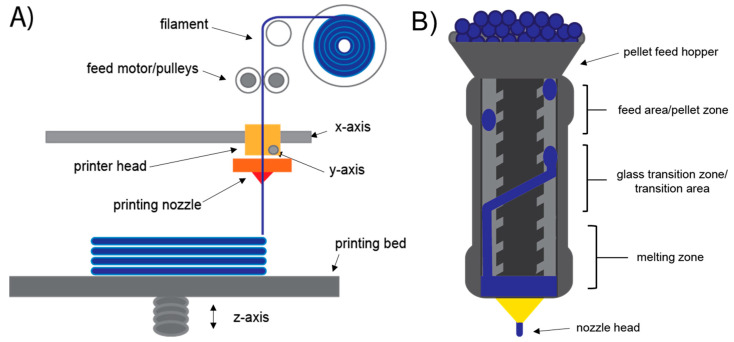
(**A**) Process of fused filament fabrication (FFF) 3D printing. (**B**) Single-screw extrusion printer head on a pellet-fed materials extrusion system.

The generic 3D printing process consists of three process steps including: (1) preparing a model of the part to be made using computer aided design (CAD), (2) separating the CAD model into “slices” using slicing software that allows the printer to process the model in three dimensional space, and (3) printing the slices sequentially using the software commands (G-codes) from the slicing software resulting in the production of the part. In this paper we will focus on the wood materials used to manufacture 3D objects through material extrusion AM processes such as fused filament fabrication (FFF) (Figure 1).

The AM processes and utilized wood and lignocellulosic-based materials are summarized in Table 1 [15,16,17,18]. The differences among these processes are related to the method of layer deposition and material type utilized. AM processes typically used with wood and lignocellulosic-based materials include extrusion-based, granular material bonding, and liquid deposition modeling. Material extrusion processes for thermoplastics and composites utilize feedstock in the form of filaments, such as FFF process as shown in Figure 1A, or pellets, as in the case of large-scale platforms. For wood and lignocellulosic-based composite feedstock materials, the most commonly used thermoplastic matrices include poly lactic acid (PLA), wood flour, spray or freeze-dried cellulose nanofibers (CNF), and lignin as the wood and lignocellulosic-based filler material [15,19,20,21,22,23,24].

In FFF systems, the thermoplastic and filler component typically needs to be compounded together prior to use in an extruder and converted into filament. The thermoplastic filament is fed using a motor into a heated printer head where it is melted and deposited using a nozzle onto the print bed. The printer head deposits the melted thermoplastic in layers (slices) in the x- and *y*-axis’s, with the printing bed moving between layers in the *z*-axis (Figure 1A). Blended pellets can also be loaded into the printer head on some pellet-fed machines and used directly without the need for filament formation. These systems utilize a screw-based extruder and will be discussed in greater detail in a later section (Figure 1B). Bioprinting is also an extrusion-based 3D printing process. In contrast to the melted thermoplastics used in traditional extrusion-based methods, bioprinting uses aqueous suspensions of nanocellulose and other natural-derived biopolymers to produce hydrogel materials. These hydrogels can be printed similarly to thermoplastics and have found use in biomedical applications such as wound dressing, drug delivery and scaffolding [14,25,26].

Granular material bonding processes such as selective laser sintering (melt bonding) also use thermoplastic feedstocks and wood and lignocellulosic-based fillers. This method utilizes a bed of powder which is melted or sintered at specific locations to form the desired shape. The printing platform is then moved downward, and additional powder is added atop to produce additional layers. It is also worth noting that there are also water activation processes using combinations of wood feedstocks and inorganic binders (gypsum, sodium silicate and Portland cement) [27,28]. Lastly, liquid deposition modeling is a hybrid extrusion-based manufacturing process which uses combinations of wood feedstocks (saw dust or wood flour) and methyl cellulose or latex-based thermoplastics, with water as a dispersant or wood adhesive as binder [29,30,31,32]. However, this article will focus on thermoplastic composites with wood and lignocellulosic-based fillers, printed on material extrusion platforms.

## 3. Wood and Lignocellulosic Composites and their Properties

### 3.1. Wood Flour, Fiber, and Sawdust

With increasing environmental and cost concerns of fillers like carbon fiber (CF), researchers working on 3D printing of composites are looking into new sustainable options. Wood materials are a low cost, biomass alternative, which have shown great promise. Additionally, many wood materials also exhibit good tensile strength and stiffness, similarly to their CF counterparts [33,34]. There are many wood species and materials being examined in 3D printing composite formulations, including those derived from softwoods and hardwoods.

Depending on the wood species, and comminution process used, wood particle size varies from 14 microns up to 2000 microns. To produce more consistent sizing, wood particles are typically segregated using a series of mesh screens. Using wood particles arising from the screening process results in a more homogenous size and better properties in the final composite. Unfortunately, in some instance’s researchers do not specify wood species or particle size in their publications making it challenging to compare the effects of these factors on the resulting material properties among studies. The wood species, particle sizes, polymer types, and loading levels commonly being explored are listed in Table 2. All samples were 3D-printed, flat on the print bed. An effort was made to find references that used similar printing techniques for the purpose of comparison. To synthesize composites, the wood particles are compounded with common polymer types, including poly-lactic acid (PLA), polypropylene (PP), styrene maleic anhydride (SMA) copolymer, polyvinyl acetate (PVAc), and urea-formaldehyde (UF). However, attributable to several considerations including degradability and ease of printing, bio-derived PLA has received much of the attention in 3D printed wood composites. PLA can also be functionalized to allow for better interaction with fillers [35,36]. Some researchers have reported using commercially available wood-filled filaments with brand names such as ColorFabb Woodfill (a blend of PLA and PHA and 15 wt.% wood fiber, ColorFabb, The Netherlands), Laywood 40% wood fiber (LayFilaments, Germany) and Easy Wood Coconut Form Futura with 40 wt.% coconut fiber (FormFutura, The Netherlands) [37].

Enhanced thermomechanical properties are a good measure of success for AM materials utilizing wood-materials. Improvement in properties can be obtained when good interactions between the polymer matrix and filler are achieved. However, when interactions are poor, resultant materials can be brittle and lack strength. Mechanical properties, including tensile strength, elongation at break, and Young’s modulus for some printed PLA composite examples are listed in Table 3 As a result of large particle size and heterogeneity in samples, incorporation of wood fillers such as sawdust and wood flours are typically kept below 20 wt.%. Higher levels of filler loading result in an inability for the polymer to surround and interact with the particles, leading to brittleness and premature mechanical failure. Kariz et al. observed this trend in their material extrusion AM composites of beech wood and PLA, where increasing wood content from 20 wt.% to 30 wt.% resulted in a decrease in both tensile strength and Young’s modulus from 49 to 48 MPa and 3.94 to 3.84 MPa, respectively [38]. Overall, introduction of wood-materials into AM components show promising properties when particle size and filler percentage are carefully considered.

### 3.2. Lignocellulosic-Components: Cellulose and Lignin

**Figure 2:**
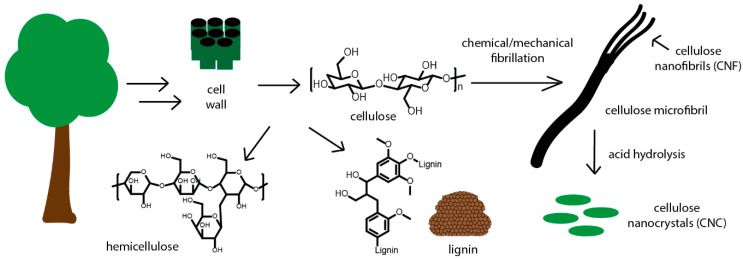
Plant cell walls comprised of cellulose, lignin, and hemicellulose. The cellulose can be further isolated and broken down into microfibrils, CNFs, and CNCs.

Wood can be broken down into its three major components; cellulose, hemicellulose, and lignin (Figure 2). These components are also found in other plants, at different ratios. In wood, cellulose accounts for around 40–50% of the total weight, while in plants like cotton fiber this amount is even higher, near 90%. Cellulose is a polymeric structure composed of repeating *β*-(1–4 linked), D-glucose linkages, which are aligned as microfibers within the plant wall [48,49]. These microfibers (microcrystalline cellulose (MCC)) can be broken down into smaller fibril bundles with diameter less than 100 nanometers. These smaller bundles, called cellulose nanofibrils (CNFs), are comprised of individual fibers which are held together through strong hydrogen-bonding [50]. Within the cellulose, there are amorphous and crystalline regions, which comprise due to the high degree of hydrogen-bonding present between the pendant hydroxyl groups. Highly crystalline forms of cellulose can be obtained by hydrolyzing the amorphous regions, which results in cellulose nanocrystals (CNCs) [34,51]. Based on differences in size, these cellulose materials find different uses in composite materials. Hemicellulose accounts for around 5–35% of the mass of wood and is comprised mostly of assorted polysaccharides [52]. This structure is heterogeneous, easily hydrolyzed into smaller sugars, and is not widely used in 3D printing. Lignin is a crosslinked phenolic polymer which serves as a support structure in plants. In wood, lignin accounts for around 20–35% of the total weight. Because of its structure, lignin is hydrophobic, rigid, and difficult to functionalize. Current research into utilizing lignin in AM processes involves direct utilization, but the extremely hydrophobic nature can make it difficult to obtain good incorporation in the polymer matrix [53].

Lignocellulosic components used in 3D printing applications (cellulose and lignin) are listed in Table 2. All samples presented were 3D-printed, flat on the print bed using similar print processing conditions. Cellulose has been investigated at both the microscale, including microcrystalline cellulose, and the nanoscale, including cellulose nanofibrils and cellulose nanocrystals. Cellulose is typically melt mixed or extrusion compounded with thermoplastic polymers such as PLA and PP. The heat and shear forces involved in these techniques can break down the structure and cause thermal degradation, so careful attention needs to be paid during compounding. Researchers have been examining the effect of surface modification on properties of printed materials. By functionalizing the hydroxyl groups on cellulose and lignin, the extreme hydrophilicity and hydrophobicity apparent in each material respectively can be overcome to increase good interactions with the polymer matrix. These good interactions result in improved mechanical properties as microphase separation is not present in the samples. Promising work involves grafting small molecules and polymers onto cellulose, which can introduce hydrophobicity and decrease aggregation [54,55,56,57,58,59,60].

Researchers have investigated addition of lignocellulosic components in a variety of thermoplastics. Material properties, specifically thermomechanical properties, are an important measure of the successful incorporation in newly developed AM materials. The variation in properties can help explain the interactions between the thermoplastic polymers and the lignocellulosic-based fillers used in each case. Tensile strength, elongation at break, and Young’s modulus for some of the PLA composite examples are listed in Table 3. Because of its biodegradable nature, PLA has received the most focus with wood materials. When good interactions between the plastic and filler occur, enhancement in mechanical properties is observed. This includes an increase in stiffness and strength, which correlates to lower elasticity, attributable to the presence of stiffer filler materials. Additionally, there is an optimal amount of filler material, similar to that observed with wood fillers, where too much filler results in mechanical failure and brittleness. Filler levels below 30 wt.% for CNFs show the most promise, with greater amounts resulting in a decrease in all three mechanical properties. Tekinalp et al. observed this with CNF where greater amounts of filler were too viscous to extrude for 3D-printing, and there was a decrease in strength and toughness with higher CNF contents [44]. Functionalizing of CNF particles using approaches such as acetylation and polymer grafting, can also result in better adhesion between the polymer and filler, but this also requires lower filler contents to be beneficial. Alternatively, for larger size lignocellulosic components like MCC and lignin, lower levels of wood materials display the best mechanical properties. However, the mechanical properties for these composites are typically inferior to those made from the smaller particles, or higher aspect ratio fillers, like CNF and CNC. Though, because of its high crystallinity, CNCs are stiffer than CNFs and need to be utilized at lower filler contents. Additionally, the aspect ratio of these materials differs greatly between cellulose sources and plays an important role in the interactions between filler and matrix, which will be discussed in the next section.

## 4. Challenges and Opportunities in 3D Printing of Wood Composites

### 4.1. Feedstock Processing

#### 4.1.1. Material Compounding

There are a few considerations that need to be addressed prior to 3D printing with an extrusion-based machine, primarily surrounding the use of filament as the printing medium itself. Firstly, the thermoplastic and wood and lignocellulosic-based components need to be adequately mixed. Traditionally, this involves melt compounding. Many of the processing steps used to compound wood and lignocellulosic components into plastics for 3D printing are analogous to the processing steps commonly used in wood plastic composites for profile extrusion, injection and/or compression molding [52,61]. The wood or lignocellulosic component furnish should be dried below 1% moisture content to facilitate proper mixing with the plastic. The filler components are typically mixed with the plastics and/or other additives via batch mixing in thermokinetic mixers or continuously using twin screw compounding extruders. Depending on component loading levels required in the thermoplastic, the creation of a masterbatch at higher filler loading level followed by dilution to lower filler loading levels can be beneficial to improved distribution and dispersion of the filler in the polymer matrix [62]. The plastics examined thus far (i.e., PP, PE, PLA) for 3D printing typically have melt temperatures below 225 °C which is below the thermal decomposition temperature of wood and lignocellulosic components. This does unfortunately limit the range of plastics applicable in wood and lignocellulosic-based materials. Researchers are looking into functionalizing wood-components to increase degradation temperatures and allow for expansion into additional plastics with higher melting temperatures such as polyamides (PA) and polycarbonates (PC), but this will be discussed later in the paper.

#### 4.1.2. Filament-Fed Systems

**Figure 3:**
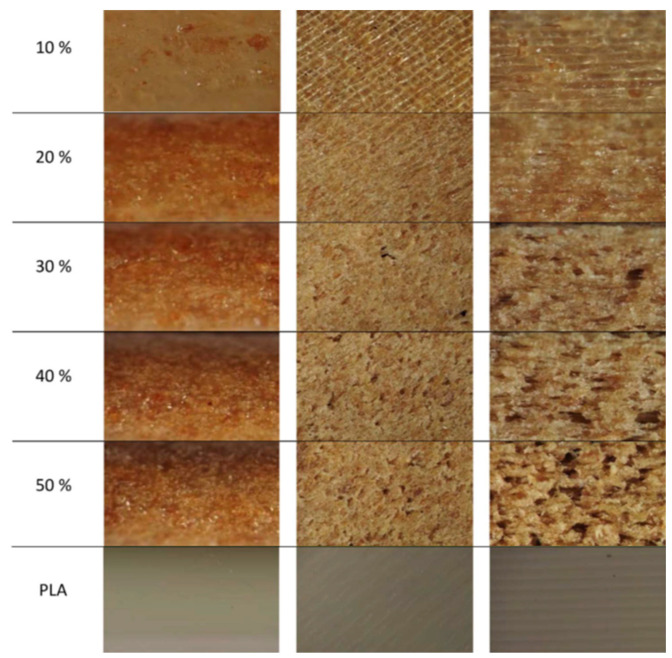
Challenges with filament production including voids, pores, and uneven distribution of filler. Cross-sectional optical micrographs displaying the appearance of filament (**left**), surface (**middle**) and edge (**right**) of the 3D printed part. Reprinted from [38] with permission from Elsevier. Copyright (2018).

Once compounded together, the next major hurdle in materials extrusion process AM printing is the conversion of the mixture into a filament. Most bench top materials extrusion process AM printers typically use filament fed extruders to produce parts. Filament fed extruders have specific requirements for filament diameter and dimensional tolerances such that improper filament size, presence of voids or shape (non-circularity) can be problematic to properly feed the printer and produce adequate parts [16]. The presence of wood and lignocellulosic-based components, even at low loading levels, can easily act as defects in the filament and result in difficulty maintaining dimensional tolerances and filament quality [63]. Higher loading levels of wood and lignocellulosic-based components can result in problems with filament brittleness and breakage as well as issues with even printing the wood-filled filament [19]. It is also very important to control the particle size and loading level of the filler being added to the plastic. High loading levels and large particles can affect the quality of the filament in terms of poor printing quality and printer nozzle plugging, an issue related to viscosity of the melted filament and pressure required to push filament through the nozzle. Issues with 3D printer nozzle plugging can be partly overcome by using nozzles with the proper size opening, although large nozzles can result in a large bead size, which cannot always be used for all applications. An example of filament production problems is shown in Figure 3, where increasing amounts of wood filler produced larger, more common filament issues (pores). Filament voids (pores) can form during the extrusion process and can be inconsistent throughout the filament. Possible causes contributing to the creation of pores include fibers incompatible with matrix polymers and nozzle clogging attributable to insufficient extrusion force [64]. Moreover, the viscosity of the fiber-filled polymer composites are increased compared to neat polymers, thus composite filaments require a larger processing force. Filament-based melt-extrusion 3D printers typically do not utilize screws in their heating barrels. The flow of melts primarily relies on the modulus of the filament, gravity of materials and the force from feeding gears trying to push the filament into the barrel [16]. If the viscosity of a polymer (i.e., PP and PE) is not sensitive to temperature change, the addition of fibers into these polymers can result in flow difficulties and deposition issues. Additionally, the filler can also become aggregated resulting in uneven distribution throughout the filament. Tailoring filler-filler and filler-polymer interactions by functionalizing the fillers can help overcome some of these filament formation and printing issues.

#### 4.1.3. Pellet-Fed Systems

Comparatively, pellet fed extruders are standard for large scale materials extrusion process AM printing. More recently, these extruders are becoming common for bench top 3D printers, as well [65]. Pellet fed extruders help circumvent some of the issues related to producing quality filament. Additionally, use of polymer pellets is a lower cost approach compared with polymer filament. Thermoplastics and fillers can be compounded together in the extruder, while alternatively pre-compounded pellets can also be used for smaller-scale pellet-fed extruders [66]. Low density materials can be difficult to feed into the system due to static, so careful feeding is imperative to prevent differences in material composition. Additionally, due to the size of the single-screw extruder, there is a size limitation for the pellets in order to obtain complete melting and mixing. A pellet fed extruder possesses a single screw, creating shear flow which can lower the viscosity of the composites to the level of pure polymers at high shear rates [22,67,68]. There is a limitation in filler wt.%, as high filler content can result in a viscosity which can clog the extruder. Pellets are pushed from the feed area into a transition area using the screw where they are melted. The size, shape, and consistency of the printed bead can be controlled by varying the screw [69]. Shear rate is important because high shear can result in backpressure, which can result in inconsistent bead size and poor surface quality [70]. Post-print finishing and milling can easily fix this issue.

Regardless of the feeding method, the filament or pellets need to be dried prior to printing to reduce possible problems with absorbed moisture during the printing process [37]. Making test prints using new wood-filled thermoplastics is a good way to screen various formulations for printing quality, especially in terms of interlayer bonding and print quality based on rheological behavior [71]. Various approaches to circumvent some of these issues will be discussed in further sections.

### 4.2. Polymer Crystallinity

**Figure 4:**
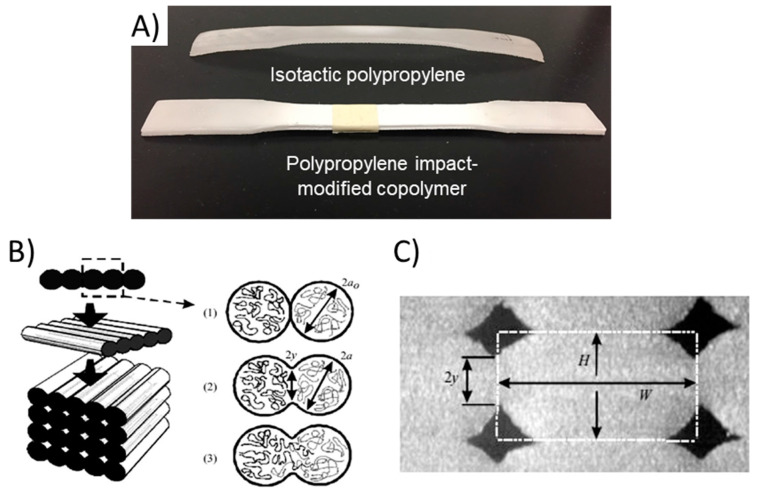
(**A**) Comparison of the dimensional stability of 3D printed PP and PP copolymer. Reprinted by permission from Springer [Journal of Thermal Analysis and Calorimetry. Ref. [64], copyright 2018] [64]. Schemes of (**B**,**C**) Bond formation process between two filaments: (1) surface contacting; (2) neck growth; (3) molecular diffusion at interface and randomization. Reprinted from [17] with permission from Elsevier. Copyright (2017).

In the past, there are three thermoplastics that have been used extensively in the wood plastic composites (WPCs) industry, polypropylene, polyethylene and polyvinyl chloride [61]. However, these three plastics have limited commercial availability for 3D printing applications. The primary reason for the limited availability of such materials in melt extrusion-based 3D printing is their dimensional instability mainly due to their high thermal expansion coefficient, usually warping caused by rapid crystallization during the printing process as shown for PP in Figure 4 [62,72]. A simple way to address the warping issue in 3D printing is to avoid using highly crystalline plastics [73]. Polypropylene copolymers, including PP block copolymer and PP random copolymer have been successfully printed with less shrinkage and warping (Figure 4A) compared to isotactic PP (iPP) [64,74,75]. Likewise, it is envisioned that syndiotactic or atactic PP could also be explored in extrusion-based 3D printing, though no results have yet been reported in the literature. Recently, low density polyethylene (LDPE) with a lower crystallinity (28%) was successfully added to high density polyethylene (HDPE) (62%) to improve the dimensional accuracy of 3D printed parts [76]. Additionally, some researchers have utilized post-print thermal annealing to overcome crystallization issues of some semi-crystalline 3D printed polymers [77,78]. The extended heating time and slow cooling allows for better control of crystallization and enhanced properties. Unfortunately, this work has not yet been expanded to use with PP or PE.

It is noteworthy that most of the thermoplastic products in the wider commercial plastics marketplace are made of polymers with high crystallinity, which often results in better mechanical properties [79]. In an effort to improve the processing of such materials and retain or enhance mechanical properties, addition of nanofibers has been investigated for 3D printing [62]. Addition of fillers in a semi-crystalline polymer can affect its crystallization behavior in different ways and at different stages of processing. During the nucleation process, most fillers can act as nucleation agents that provide additional sites for spherulitic growth. However, during the crystallization growth stage, the gaps among fibers are typically extremely narrow, thus crystal growth can be limited within the small spaces [62]. Overall, nanofibers act better as crystallization retardants, instead of nucleating agents for semi-crystalline polymers [80]. The fiber loading level of nanofibers should be carefully controlled to regulate the crystallization behavior of the polymers during processing allowing for better control of crystal growth.

Crystallinity also plays an important role in the processing of composite materials. To be properly processed, highly crystalline polymers need to be completely melted, which requires processing well above melting temperatures and can be quite high for some highly crystalline polymers (polyamides). These high temperatures can result in decomposition of the wood fibers and limit use with certain plastics. This also means that higher printing speeds and feed rates can result in incomplete filament melting leading to greater crystallinity and inferior printed materials [81]. Printing head temperature can be changed to help alleviate this, but increased nozzle temperatures can result in other issues in the final printed material. Temperature of the printing bed can also be adjusted to help control crystallization and provide less warping and shrinkage of printed parts [82,83]. Overall, each process step of extrusion-based printing needs to be controlled as it can affect crystallinity and thus mechanical properties of the resulting materials.

### 4.3. Interlayer Adhesion

There are differing opinions over whether the addition of fibers degrades or enhances the interlayer bond strength of 3D printed parts. Interlayer adhesion relates to the mechanical anisotropy between deposited beads during printing. [84]. During printing (i.e., melting and depositing polymer), the shear forces lead to high orientation. Unfortunately, this can result in poor diffusion of polymers and thus poor entanglement between polymer chains in various layers, resulting in poor interlayer adhesion and thus poor properties. On the one hand, fibers remaining at the interface between two layers can reduce the area for polymer chains to diffuse from one side to the other [85]. Because interlayer bond strength is proportional to the degree of polymer diffusion, and this can be reduced by the presence of fibers, such strength can be reduced by the presence of fibers. Alternatively, if the interaction between the fibers and polymer matrix is strong, e.g., with the application of coupling agents or other functionality, stress can be transferred from the polymer chains to the fibers, resulting in increased interlayer bond strength (Figure 4B) [17]. In 3D printing, most fibers will align in the printing direction attributable to the extrusion process. The strength properties of a printed part along the fiber’s transverse direction is often weaker than in the longitudinal direction. To improve the interlayer bond strength, fibers will need enhanced transverse strength. Additionally, a large degree of crystallization was found to degrade the interlayer bonding strength of semi-crystalline polymers [86]. When each laid-down polymer strand cooled, it experienced shrinkage, and tended to pull itself away from adjacent strands, creating extra separating forces at the interface (distance 2y in Figure 4C). The proper addition level of nanofibers with adequate transverse integrity can reduce the matrix crystallinity and provide reinforcement to polymer chains near the interfacial area, and therefore, potentially increase the interlayer bond strength. Additionally, it has been shown that print processing conditions can affect interlayer adhesion for printed thermoplastic matrices [87]. Increasing nozzle and bed temperatures can allow for better diffusion, resulting in great interlayer adhesion. Therefore, controlling printing conditions can result in enhanced thermomechanical performance, allowing for additional control over interlayer adhesion in 3D-printed composites.

### 4.4. Printing Orientation

Printing direction plays an important role in final part material properties. Variation in printing orientation can result in weak areas within the material, leading to premature failure. The orientation in which a material is printed can be control in two ways; orientation on the printing bed and direction of polymer infill [88,89]. Liu et al. studied the effect of printing bed orientation on the mechanical properties of AM-printed wood-filled PLA composites [90]. Composites printed upright results in the worst properties attributable to poor interlayer adhesion associated with transverse printing patterns. Overall, flat and on-edge samples displayed good interlayer adhesion, consistent with longitudinal printing, which allowed for good dispersive force, resulting in mechanical properties consistent with virgin PLA.

**Figure 5:**
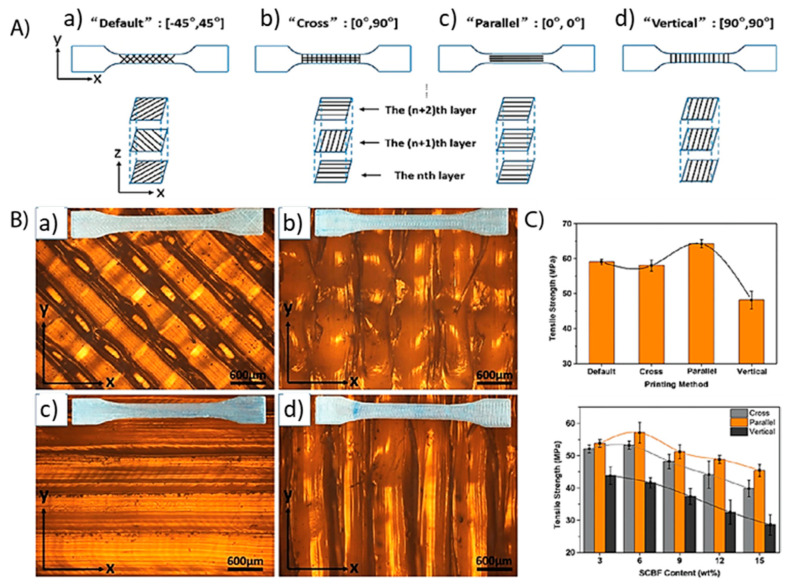
Effect of infill and printing orientation patterns including (a) default, (b) cross, (c) parallel, and (d) vertical. (**A**) Graphical representation of these printing orientation of infill layers. (**B**) Infill pattern images using atomic force microscopy (AFM). (**C**) Tensile strength of PLA composites printing using various infill patterns. Reprinted from [91] by permission from John Wiley and Sons. Copyright (2019).

Infill pattern accounts for how the inside of a printed sample is filled. There are multiple parameters associated with this including the direction of printing versus the sample, whether this direction changes with each layer, and how much of each layer is filled in (fill density). Each layer can be printed parallel or perpendicular (or other angles) relative to the sample length. Layers can also be alternated in direction to create patterns of infill, such as the default (x-shape) or cross shown in Figure 5. The fill density can also be controlled, however less density typically results in more voids and inferior properties. When it comes to the effect direction of printing for each layer, Liu et al. found that mechanical properties are correlated directly with printing direction [91]. Better tensile strength and stiffness were observed when layers were printed in parallel, meaning that the layers (and each strand) are printed parallel to the direction of stress (force). This results in good interlayer adhesion, which allows for better stress dissipation and accounts for the property increase. The opposite was true for samples printed vertically, in which layers and strands are perpendicular to the direction of stress. In dog-bone samples used for tensile testing, this results in short strands that have poor adhesion. The resulting mechanical properties are the worst of all printing directions. Alternatively, samples which featured alternating layers, resulting in a cross or x-shape pattern both displayed good adhesion. However, the overlapping points between layers undergo high stress during mechanical testing and typically these infill patterns contain more voids, which results in mechanical failure and inferior properties to the superior parallel studies. To obtain good properties from 3D-printed materials, care must be taken to choose a printing direction and infill pattern consistent with good adhesion and minimal defects in the final part.

### 4.5. Filler Dimensions (Aspect Ratio)

The shape of the fillers is important to the final properties in the composite [92,93]. Wood materials come in a variety of particle shapes, for easier comparison to each other, characterization such as aspect ratio is used to normalize shapes versus each other. Aspect ratio provides a ratio of length to width for a particle. Small aspect ratios are typical for shapes where length and width are similar, such as spheres. Alternatively, large aspect ratios are found in thinner fibril (rod) shapes, where particles feature longer lengths and shorter widths. Certain wood and lignocellulosic components have a higher aspect ratio than others. Cellulose nanofibrils have an aspect ratio ranging from 25–500, because of the long length (microns) compared to short width. Comparatively, cellulose nanocrystals have much smaller aspect ratios anywhere from 10–100 maximum, attributable to much shorter lengths (<500 nm) and comparative width. Similarly, large particles like wood fibers and microcrystalline cellulose have aspect ratios typically less than 100, however these particles are very large in all dimensions comparatively (microns for both length and width) [48,94,95,96].

**Figure 6:**
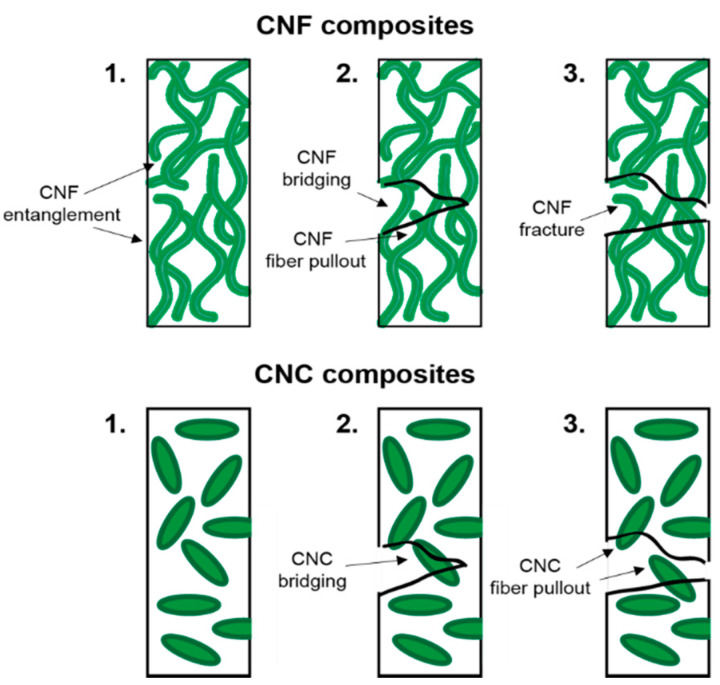
Illustrations of fracture mechanisms of CNC and CNF nanocomposites; (**1**) before stress, (**2**) during mechanical stress, and (**3**) after stress. Longer CNF fibrils can bridge the fracture surface, resulting in a stronger composite.

When mixed into a polymer matrix, the filler dimensions have a large effect on microstructure of the composite [97,98]. The long fibers of CNF have a large surface area and length. As interlayer adhesion is key to mechanical properties of AM parts, it is beneficial to have fibers long enough to be able to bridge between printed layers. Alternatively, in shapes like spherical/oblong CNCs, the particle length is shorter, and the particles are not typically able to diffuse or entangle between printing layers. The shape also results in less interfacial area between the particles and matrix, resulting in potentially poorer interactions and inferior properties [99]. Additionally, other fillers with a higher aspect ratio can bridge within the polymer matrix to better dissipate stress, attributable to increase entanglement with the polymer matrix (Figure 6).

### 4.6. Large-Scale Printing

Large-scale printers are a relative newcomer to AM and are helping voyage the path between lab-scale experimentation and industrial production capabilities. Whereas typical 3D-printers are benchtop size (average of~8 cubic centimeters), these large-scale printers typically possess build volumes of 1 cubic meter or larger, with some systems like the big area additive manufacturing (BAAM) printer from Cincinnati Incorporated (Harrison, OH, USA) occupying a much larger build volume of 35.5 cubic meters [100]. As mentioned earlier, these systems utilize a pellet-fed extruder.

Due to its size, there are some unique challenges associated with large-scale printing. Temperature control, print time, and resolution go hand-in-hand. Higher printing speeds or large bead sizes can be used to decrease printing time, but these often result in poor resolution on the final product [101]. Another consequence of the longer printing times and large volume is potentially difficult to control temperature gradient. This can be detrimental to the final product as temperature is important to ensure good interlayer adhesion, prevent warping, and insure homogeneous printed filament. With the incorporation of biomass-based fillers, these issues are even more common [102]. The presence of wood and lignocellulosic-based fillers can results in poor adhesion due to a lack of polymer entanglement, crystallization problems, and heterogenous dispersion, which were all discussed previously in their relevant sections. To overcome these issues, large-scale printers rely on tunable screws during the extrusion, heated beds, dual-printing heads, and environmental control, as well as extensive trial and error using test prints [100].

As both large-scale printers and use of wood and lignocellulosic-based fillers are more recent advancements to materials extrusion printing, there is little research done yet involving both. Currently, the only published research on large-scale printing with wood and lignocellulosic-based components comes from Zhao et al. They used popular fibers, incorporated into a PLA matrix, to print architectural pieces and found that careful adjustment in printing processes, combined with use of selective fiber size, resulted in controlled viscosity and successful printing [103]. Despite the challenges of large-scale printing, researchers are finding new ways to overcome and excel at this novel methodology. More work is needed to expand into the use of wood and lignocellulosic-based materials. With its cheaper, sustainable draw, there is a strong future for large-scale extrusion printing of wood and lignocellulosic-based components.

## 5. Applications

Materials extrusion AM processes have evolved over the last decade to allow these materials to encompass many applications. High performance materials for construction, tooling, automobiles, recreation equipment, and even biomedical devices can now be produced using materials extrusion. However, many of these materials are traditionally printed using petroleum-based chemicals. One of the most common polymer matrices for many of these applications remains acrylonitrile butadiene styrene (ABS), which is often filled with carbon fiber or glass materials. Replacing any of these components with a sustainable alternative proves a promising approach. Of these, the easiest swap remains exchanging the carbon fiber and glass with wood and lignocellulosic-based components such as wood flours and cellulose materials. These sustainable filler alternatives offer similar high strength and stiffness to their petroleum analogues, while remaining cost effective. Two areas of AM composite applications will be discussed, direct and indirect applications. Direct applications utilize the printed material as an end product, while indirect applications use the printed material as a basis to produce another product (mold, scaffolds).

### 5.1. Direct Uses

The major applications for direct use of materials extrusion printed wood and lignocellulosic composites include marine, building, transportation, and tooling applications. In these areas, products can be directly printed allowing for complete customization and quick production time. Currently, these applications rely on printing proof-of-concept smaller materials, with the long-term goal of scaling towards commercial-sized products.

**Figure 7:**
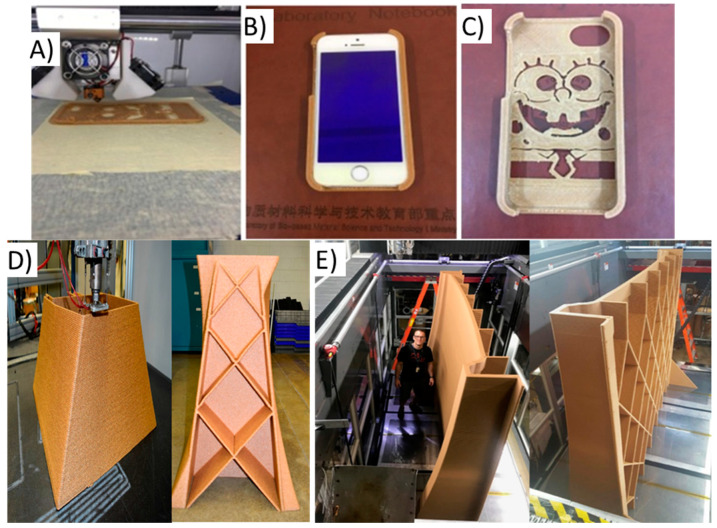
Materials extrusion AM printed direct use products. (**A**–**C**) Mobile phone shell using a wood flour composites. Reprinted from [39] with permission from Elsevier. Copyright (2018). (**D**) Building material printed using a poplar/PLA composite. Adapted with permission from [103]. Copyright 2019 American Chemical Society. (**E**) Large scale 3D printed boat roof tooling mold made from 20 wt.% wood flour and 1 wt.% cellulose nanofibrils (CNF) in a poly lactic acid (PLA) matrix [71].

In terms of smaller scale (proof-of-concept) direct applications, materials extrusion AM can be used to manufacture a multitude of products. Researchers have begun utilizing their designed wood and lignocellulosic-based composite filaments in exchange for traditional petroleum filaments in commodity products, towards using these replacements on an industrial and household 3D-printing scale. In terms of commodity products, Bi et al. used aspen wood flour/TPU composites, strengthened using compatibilizers, to print a flexible cell phone case (Figure 7a–c) [39]. Gkartzou et al. and Tanase-Opedal et al. printed cell phone cases using their separate lignin filler composites [15,47]. These materials are easily customized and can be printed easily on a multitude of scales. Similarly, Tekinalp et al. and Guo et al. used PLA composites, with CNFs and poplar wood flour respectively, to print prototype furniture [44,104]. While both were printed at a very small scale (<100 mm length), the combined structural control and mechanical properties are promising for future furniture applications. Additionally, Depuydt et al. has shown that tooling items can also be printed, such as brake levers for bicycles [105]. Production of robust, customized tools with a quick turnaround could be game changing for this industry. Overall, these small proof-of-concept printing examples show that wood and lignocellulosic composites can easily be tailored towards use as a commercial filament for use in a range of direct use products.

Alternately, commercial applications for these materials are also being explored industrially. A common example of this is use of materials extrusion printing to produce building materials. Zhao et al. printed prototype building materials using a poplar/PLA composite (Figure 7d) [103]. This material showed good printability and customization control. The low-cost of these materials and ease of production show promise for materials extruded wood and lignocellulosic composites to be used as a sustainable, low-cost option to produce building materials for economically disadvantaged areas. While the step towards large-scale printing has been taken, future research is needed to develop these materials towards commercial applications.

### 5.2. Indirect Uses

There has been extensive research involving the use of materials extrusion AM for indirect applications. One example, which is typically used at a large-scale, involves the printing of molds for various applications, including concrete casting molds, automotive part molds, and boat hull molds [101,106,107]. Materials extrusion AM printing of molds is a cost-effective method and allows for easier customization. Typically, these molds are printed on large scale materials extrusion printers due to their dimensions. This technique has been recently used to demonstrate production of a marine tooling mold using wood-based feedstocks with PLA (Figure 7e) [71]. A 3D printed boat roof tooling mold made from 20 wt.% wood flour and 1 wt.% cellulose nanofibrils (CNF) in a poly lactic acid (PLA) matrix was printed. Traditional marine molds involve a multi-step construction process which uses a variety of nonrenewable materials including steel framing, plywood sheathing, and polystyrene covered epoxy fiberglass, making it a difficult and costly process to produce multiple mold options. Comparatively, materials extrusion AM printed molds are produced through a facile process and can be easily used to cast other materials. Additionally, these printed wood and lignocellulosic composite molds can be reused multiple times or recycled and used to reprint alternative products.

Other promising indirect applications for these extrusion-printed composites includes biomedical applications, such as the printing of a tissue scaffold. As a proof of concept, Koo et al. printed dog bone samples comprised of BTPE with CNCs [42]. These samples were then used both in vivo (grow L-929 cells in a petri dish) and in vitro (implanted samples into mice). Both studies showed these materials were biocompatible, due to the combination of thermoplastic elastomer (TPE) and lignocellulosic source, and capable of supporting cellular adhesion. Traditionally, TPEs cannot support cells, but the unique conditions of materials extrusion printing impart lower crystallinity and a uniform surface for growth. This study is very promising towards the use of these materials to print entire, biocompatible structures for both in vivo and in vitro tissue engineering. Researchers are continuing to find other promising indirect applications which exploit both these materials and this technique. Overall, researchers are still exploring additional indirect use applications for materials extrusion AM wood and lignocellulosic composites.

## 6. Future Trends and Outlook

There is a promising future for materials extrusion AM with wood and lignocellulosic composites. With a global thrust towards use of sustainable materials due to environmental impacts, commercial production of sustainable materials is becoming more common. Finding new materials to commercialize and uses for these commercial materials will help push the field. However, in order to fully exploit this new field, researchers need to focus on moving from proof-of-concept applications into production of large-scale, commercial, direct and indirect use products. To accomplish this, researchers need to produce composites, as mechanically robust as traditional materials, for a wider array of applications. This will result in replacement of the traditional petroleum chemicals, currently holding the majority footprint, used in industry with many of these sustainable analogs. There are two major trajectories of focus for researchers to achieve this goal.

Firstly, researchers need to continue to develop new methodologies and optimize current technologies to better exploit optimal properties of current fillers. Research focused on methods to overcome the issues associated with materials extrusion AM of these materials, as discussed in the previous sections, would prove beneficial. For example, unique treatments and functionalization of the filler have been shown to improve polymer-filler interactions and produce extreme improvements in mechanical properties [97]. More research into of this type of technology to optimize materials extruded composites could prove game changing.

Secondly, researchers need to focus on tailoring material properties towards specific applications. For example, exploiting mechanical strength, low thermal conductivity, and durability of composites for building material applications. While development of sustainable composites towards general direct use, such as simple household items like cell phone cases, is promising, production of customized composite materials tailored towards specific applications would create a greater overall impact in the AM field. Overall, as long as researchers continue to both develop wood and lignocellulosic composites and manufacture facile direct and indirect use products with these materials, the future remains bright.

## 7. Conclusions

Additive manufacturing utilizing wood and lignocellulosic-based components has been explored for materials extrusion process AM-printing. Various combinations of wood and polymer were examined. Within wood feedstocks, various species, particle sizes from micron to millimeter length scale, and loading levels within a matrix were evaluated. Lignocellulosic components, such as cellulose and lignin, were also explored in additive manufacturing. An evaluation of the mechanical and physical properties available were analyzed for these materials, providing insight into the effect of various processing components. Challenges in AM with wood and lignocellulosic-based materials continue and include processing issues during compounding and filament production, and extrusion. Additionally, considerations regarding polymer crystallinity, dryness, printing orientations, and filler dimensions provide insight into optimizing 3D printed wood materials with enhanced properties. The future of AM using wood and lignocellulosic-based components is promising and hold many opportunities for producing lighter weight, lower cost composite parts for many direct and indirect applications.

## Figures and Tables

**Table 1:** Additive Manufacturing processes utilizing wood and lignocellulosic-based materials.

Additive Manufacturing Process	Primary Feedstock (Thermoplastic Matrix)	Wood/Lignocellulosic Component(s)	Other Additives
Extrusion-Based Fused Filament Fabrication Bioprinting	Thermoplastics (PLA, ABS, PP, nylon, polyurethane) Hydrogel material (collagen, carrageen) or nanocellulose in aqueous suspension	Wood flour, spray or freeze-dried cellulose nanofibers, microcrystalline cellulose, lignin	Pigments, coupling agents, lubricants, glass, carbon
Granular Material Binding Selective Laser Sintering Water Activation	Thermoplastics, Cementitious materials (Gypsum, Sodium silicate, Portland Cement)	Wood flour, Wood chips, lignin	Water
Liquid Deposition Modeling	Thermoplastic emulsion Resin	Sawdust, Wood Flour, methyl cellulose	Water

**Table 2:** Wood-based, including wood flours, sawdust, and barks, and lignocellulosic fillers used in materials extrusion AM processing. All samples were printed flat on the print bed with a default (±45) infill pattern unless otherwise noted.

Filler Component	Particle Mesh Size	Particle Size (Microns)	Polymer	Loading Level (wt.%)	Additives/Filler Treatment	Printing Temperature (^o^C)	Nozzle Opening (mm)	Layer Thickness (mm)	Infill (Amount, Pattern)	Mechanical Testing ^f^	Ref.
Osage Orange (*Maclura pomifera*)	230	≤63	PLA	12.5	Dried distillers’ grain ^a^	220	0.4	0.34	100%	T	[19]
Paulownia (*Paulownia tomentosa*)											
Beech (*Fagus sylvatica*)	~60	≤237	PLA	0–50	Milled	230	0.4	0.19	100% (Square)	R, FT	[38]
			PVAc/UF (adhesives)	7–87.5	Milled		3	2	85%	B	[29]
European Softwood	~200	75 (median)	UF	13	Hardener 2545 ^d^	21	1.6	2		T, F	[30]
Aspen (*Populus sp*)	100	150	TPU	0–40	PEG 6000, chitosan and MDI^e^	185				T, R	[39]
Cork (*Quercus suber L.*)		27–733	PLA	0–50	Tributyl citrate	230	0.8	0.4	100%	T, I, DMA	[40]
MDF Furniture Waste	~200	80	PLA	10 to 40 (vol. frac.)	Milled	185	0.5	0.15	25–100%		[37]
Microcrystalline cellulose (MCC)			PP/PE	0–10	Silane^g^	190	1.75	0.2	100%	FT, DMA	[41]^h^
			PLA	1,3,5	Titanate	165–190		1.55^i^	60%	FT, DMA	[20]^h^
Cellulose Nanocrystals (CNC)			BTPE^j^	0–60	Polymer-grafted	178	0.4	0.42	20%,100%	T, R	[42]
			PVOH	0–10	MCC acid hydrolysis	230	0.35	0.2	100%	T, DMA	[43]
Cellulose Nanofibrils (CNF)			PLA	0–30		180–215	0.4	0.2	100%	T, DMA	[44]
			PHB	0–3		75			100%	T	[45]
			PLA	0–5	Grafted with PLA	165	1.75			FT	[46]^k^
Pine Kraft Lignin			PLA	0–15		205	0.2–0.4	0.1	100% rectilinear	T, FT	[15]
Softwood Lignin			PLA	0–40		205–230	0.4		100%	T	[47]
Organosolv Lignin			Nylon 12	20–40	Carbon fiber added	210	0.5	2.5	100%	T, R, DMA	[24]
Organosolv Lignin Softwood Kraft			ABS, HIPS, Nylon 12	40–60	Carbon fiber added	210	0.5	2.5	100%	T, R, DMA	[23]

^a^ Used to extract residual oils using hexane. ^b^ Used wood pulp. ^c^ Used wood flour. ^d^ Commercial hardener for UF (Glues Direct, UK). ^e^ PEG 6000: polyethylene glycol 6000, MDI: diphenyl methylpropane diisocyanate. ^f^ Tensile (T), rheology (R), bending (B), flexural (F), dynamic mechanical analysis (DMA), filament tensile testing (FT), impact (I). ^g^ Contained compatibilizer SCONA TPPP 9212 GA (0.6 wt.%, BYK-Chemie GmbH, Germany), based on PP functionalized with maleic anhydride, and the processing stabilizer Add-Vance TH 130 (1.7 wt.%, Addcomp Holland BV, The Netherlands). ^h^ Printed filaments and 3D prototype part. ^i^ Thickness of the final printed filament. ^j^ BTPE: bioplastic thermoplastic elastomer comprised of 2,5-furandicarboxylate, 1,4-cyclohexanedimethanol, and poly (tetra methylene ether) glycol. ^k^ Only filaments were tested, no 3D-printed prototypes were tested.

**Table 3:** Mechanical properties of wood and lignocellulosic filled poly(lactic acid) composites collected using tensile testing data from selected references.

Polymer	Filler	Filler Amount (%)	Tensile Strength (MPa)	Elongation at Break (%)	Young’s Modulus (GPa)	Ref.
PLA	Beech (*Fagus sylvatica*)	0 10 20 30 40 50	55 ± 4.3 57 ± 1.1 49 ± 3.3 48 ± 5 42 ± 4.3 30 ± 4.4		3.27 ± 0.38 3.63 ± 0.5 3.94 ± 0.24 3.84 ± 0.55 3.86 ± 0.54 3.00 ± 0.56	[38]
	Cork (*Quercus suber* L.)	0 5 10 15 20 25 30 50	60.03 ± 2.34 38.3 ± 0.6 26.1 ± 0.5 21.9 ± 1.4 19.4 ± 2.7 17.2 ± 3.5 15.5 ± 2.5 10.4 ± 1.0	1.5 ± 0.01 0.8 ± 0.2 0.8 ± 0.2 0.6 ± 0.2 0.8 ± 0.2 0.6 ± 0.2 0.6 ± 0 0.2 ± 0	3.344 ± 0.0967 2.815 ± 0.127 2.111 ± 0.173 1.701 ± 0.167 1.455 ± 0.0731 1.375 ± 0.199 1.179 ± 0.234 0.985 ± 0.0743	[40]
	Cellulose	0 10 20 30	53.2 ± 0.3 64.8 ± 1.1 73.7 ±1.3 80.6 ± 0.9	2.97 ± 0.48 2.36 ± 0.31 1.94 ± 0.25 1.51 ± 0.16	3.19 ± 0.06 4.86 ± 0.23 6.01 ± 0.26 7.09 ± 0.41	[44]
	nanofibrils (CNF)	0 1 3 5	26.1 ± 7.8 28.3 ± 3.6 43.4 ± 1.8 34.4 ± 6.1		2.650 ± 0.0784 2.939 ± 0.168 3.407 ± 0.0995 3.076 ± 0.0958	[46]
	Pine Kraft lignin	0 5 10 15	55.9 ± 0.6 50.3 ± 0.9 50.1 ± 0.5 41.3 ± 0.5	4.6 ± 0.22 2.8 ± 0.1 2.3 ± 0.17 1.9 ± 0.34	2.30 ± 0.04 2.33 ± 0.05 2.41 ± 0.06 2.39 ± 0.06	[15]
	Softwood lignin	0 20 40	58.45 ± 0.55 39.35 ± 1.05 45.65 ± 0.05	2.5 ± 0.1 1.8 ± 0.1 1.9 ± 0.08	2.89 ± 0.014 1.46 ± 0.156 2.695 ± 0.148	[47]
